# CT-based radiomics for prediction of response to neoadjuvant immunochemotherapy in patients with esophageal carcinoma

**DOI:** 10.3389/fonc.2025.1511691

**Published:** 2025-05-12

**Authors:** Peng Zhao, Xianhe Qiao, Yikang Geng, Yaoyi Yv, Ruiqing Meng, Xiaowei Wu

**Affiliations:** ^1^ Department of Nuclear Medicine, Cancer Hospital of China Medical University, Liaoning Cancer Hospital and Institute, Shenyang, China; ^2^ School of Intelligent Medicine, China Medical University, Shenyang, China; ^3^ Department of Biomedical Engineering, China Medical University, Shenyang, China; ^4^ Department of Infectious Disease, The First Hospital of China Medical University, Shenyang, China

**Keywords:** esophageal carcinoma, neoadjuvant therapy (NACT), computer tomography, radiomics, therapeutic response

## Abstract

**Background:**

In order to investigate the value of radiomic features derived from enhanced computed tomography (CT) for assessment of therapeutic efficacy in patients with Esophageal squamous cell carcinoma (ESCC) underwent neoadjuvant immunochemotherapy (NICT).

**Methods:**

The primary cohort of this study included 120 ESCC patients who received NICT from April 2020 to August 2023, comprising 52 patients with good responders (GR) and 68 patients with non-good responders (non-GR) after NICT, the external validation cohort included 30 patients from another hospital, comprising 14 patients with GR and 16 patients with non-GR after NICT. Features were derived from both the intra-tumoral and peri-tumoral regions of the tumor in the enhanced CT image, and the least absolute shrinkage and selection operator (LASSO) regression was used to establish predictive radiomic models (Rad-Scores) and T-stage model for predicting therapeutic response to NICT.

**Results:**

The Rad-Score for predicting response to NICT generated the area under the curve (AUC) values of 0.838, 0.831, and 0.769 in the training, internal validation, and external validation cohorts, respectively. For T-stage, corresponding AUC values were 0.809, 0.800, and 0.716 in the same cohorts. Additionally, the nomogram model produced AUC values of 0.867, 0.871, and 0.818 in the training, internal validation, and external validation cohorts, respectively.

**Conclusions:**

The established models demonstrate promising predictive potential for assessing the efficacy of NICT in ESCC patients, which may assist clinicians in formulating appropriate treatment strategies.

## Background

Esophageal carcinoma (EC) is one of the most common malignant tumors globally (1). Esophageal squamous cell carcinoma (ESCC) counts for 90% of all esophageal carcinomas worldwide, while esophageal adenocarcinoma (EAC) comprises 10% (2). ESCC patients generally have a poor prognosis, with 5-year survival rate is only 20% (3). In Western Europe, the incidence rates of ESCC in men and women are 6.6% and 1.8% (4). Neoadjuvant chemotherapy or neoadjuvant chemoradiotherapy combined with radical esophagectomy has gradually replaced surgical treatment, becoming the standard treatment for locally advanced ESCC. Neoadjuvant therapy refers to certain treatments administered before curative treatment to reduce recurrence rate by eliminating micrometastases and it increases the success rate of subsequent surgeries (5, 6).

However, during the course of radiotherapy, there may be an increase in surgical complexity and the onset of related complications such as radiation pneumonitis and esophageal fistula, leading to a decrease in patients’ quality of life. This could ultimately be a contributing factor to the poorer prognosis of ESCC patients. The incidence of local or distant metastases remains high, necessitating the further development of a novel adjuvant treatment regimen (7). Immunotherapy combined with chemotherapy offers a new strategy for patients with esophageal squamous cell carcinoma. Studies have shown (8, 9) that compared to neoadjuvant chemotherapy and radiotherapy, this treatment significantly reduces the difficulty of surgical resection, leading to overall good postoperative recovery in patients. Therefore, preoperative assessment of response to neoadjuvant immunochemotherapy (NICT) is crucial for devising appropriate individualized treatment plans.

Radiomics is a technique that transforms medical imaging data into exploitable quantitative information through statistical and machine learning methods (10), which enables objective and comprehensive assessment of tumor heterogeneity (11). In the past few years, CT imaging has played an irreplaceable role in cancer detection, screening, and treatment efficacy assessment (12). Currently, enhanced CT serves as a crucial tool for assessing the initial staging and treatment response of ESCC patients. Whether radiomic information based on enhanced CT can predict the efficacy of NICT for ESCC remains unknown. Furthermore, previous radiomic studies on ESCC (13–17)have primarily focused on intra-tumoral regions, with little attention paid to peritumoral radiomics features. Therefore, we have included peri-tumoral regions of the tumor, attempting to find potentially valuable information.

This study aims to develop a radiomic nomogram based on enhanced CT, integrating intra-tumoral and peri-tumoral radiomic features with clinical predictive indicators to identify good responders (GR) and validate its performance on external validation cohort.

## Methods

### Patients

The retrospective study was approved by the hospital’s Ethics Committee (No.XJS20230632), and the requirement for informed consent from patients was waived. We retrieved the hospital records of patients admitted from April 2020 to August 2023, and a total of 120 patients were included in this study. And external validation was conducted by including 30 patients from another hospital during the same period. Patients were categorized into GR and non-GR groups, with GR and non-GR including tumor regression grade (TRG) 1–2 and TRG 3-5, respectively.

**Figure 1:**
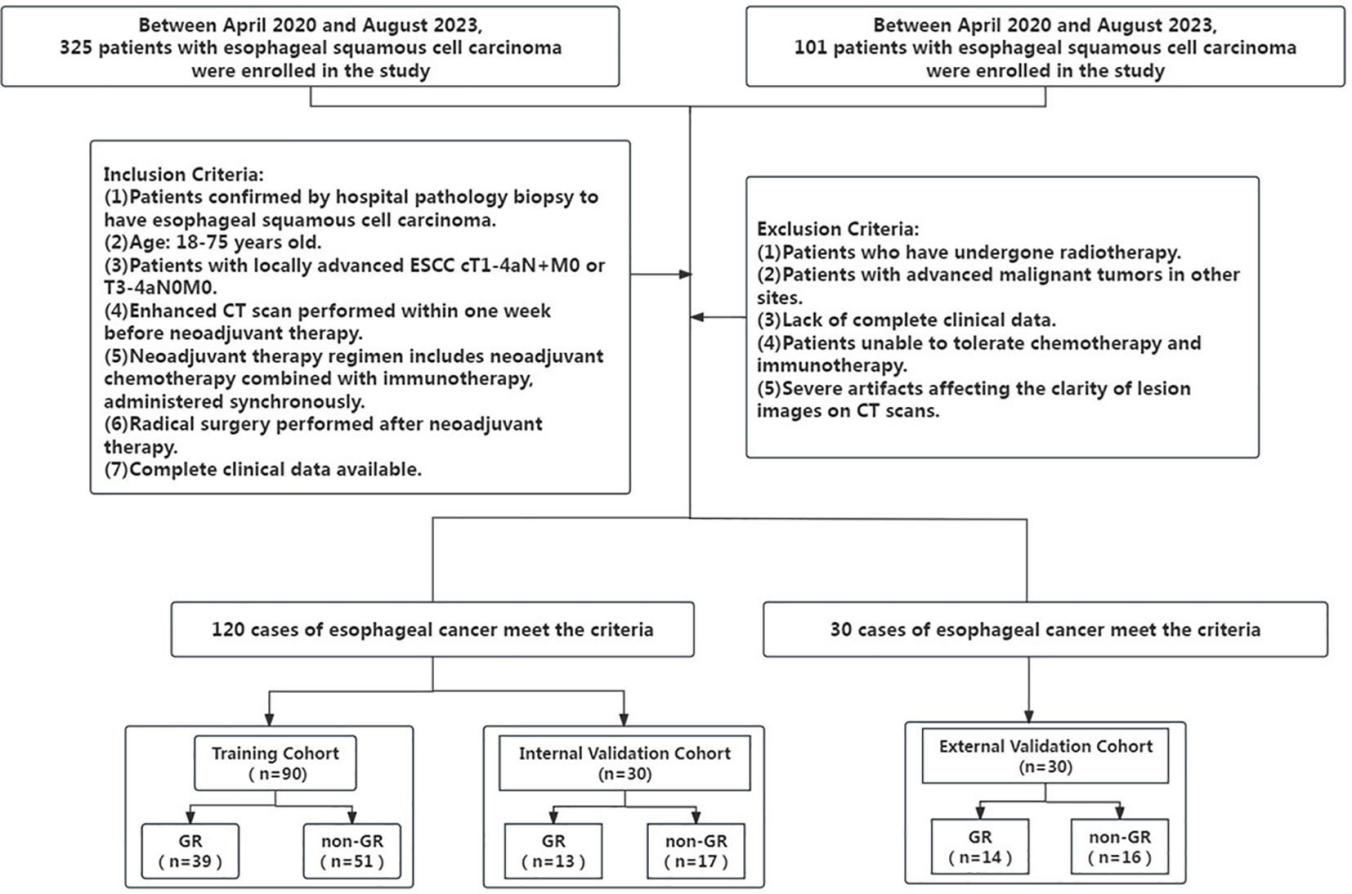
Patient inclusion and exclusion process diagram.

Inclusion criteria: (1) Patients confirmed by hospital pathology biopsy to have esophageal squamous cell carcinoma; (2) All included patients were of Han Chinese ethnicity, and age 18–75 years old; (3) Patients with locally advanced ESCC cT1-4aN+M0 or T3-4aN0M0; (4) Enhanced CT scan performed within one week before neoadjuvant therapy; (5) Neoadjuvant therapy regimen includes neoadjuvant chemotherapy combined with immunotherapy, administered synchronously; (6) Radical surgery performed after neoadjuvant therapy. (7) Complete clinical data available. Exclusion criteria: (1) Patients who have undergone radiotherapy; (2) Patients with advanced malignant tumors in other sites; (3) Lack of complete clinical data; (4) Patients unable to tolerate chemotherapy and immunotherapy; (5) Severe artifacts affecting the clarity of lesion images on CT scans. **Figure 1** depicts t Patient inclusion and exclusion process diagram.

All patients were subjected to stratified sampling and randomly divided into training and validation groups at a ratio of 2:1. Clinical characteristics and pathological information included age, gender, tumor location, Clinical T stage, lymph node N stage, metastasis M stage, pathological diagnosis, clinical stage, and number of treatment cycles.

### Tumor regression grading criteria

The Mandard standard (18) was the earliest standard used for postoperative TRG in ESCC treatment. According to the relationship between residual tumor cells under the microscope and fibrosis response, the Mandard standard divides tumor regression response into 5 levels: TRG level 1=no residual cancer cells, replaced by a large amount of fibrosis; TRG level 2=scattered cancer cells in fibrosis; TRG level 3=more fibrosis than residual cancer cells; TRG level 4=less fibrosis than residual cancer cells; TRG level 5=no tumor regression changes. The pathological response is evaluated by pathologists specializing in ESCC, and any cancer cells that do not survive in all excised specimens are referred to as GR. Patients with TRG scores of 1 and 2 are classified as good responders. Patients with TRG levels of 3, 4, and 5 are defined as non-good responders.

### CT data acquisition and tumor segmentation

Before underwent NICT, patients in the training and internal validation cohorts underwent chest contrast-enhanced scans using the Netherlands Philips IQon spectral CT, and patients in the external validation cohort underwent contrast-enhanced CT scans using the Netherlands Brilliance iCT 256 (Philips Healthcare). The scan parameters were as follows: tube voltage of 120 kVp, automatic tube current modulation (RightDose), pitch of 1.016, detector collimation of 0.625 mm × 64, gantry rotation time of 0.5 s for one revolution, matrix size of 512 × 512, and reconstructed slice thickness of 5 mm. An iodinated contrast agent (300 mg/mL) was administered at 1.5 ml/kg body mass at a 2 mL/s rate.

In this study, we utilized Pyradiomics (version 2.1.0) for feature extraction, R (version 4.2.0) for statistical analysis and modeling, and Python (version 3.7) for additional data processing. The computational tasks were performed on a workstation equipped with a 12th-generation Intel Core i9–12900 processor (CPU, base frequency 2.4 GHz), 64GB of memory (RAM), and an NVIDIA GeForce RTX 3090 Ti graphics processing unit (GPU). All images were adjusted according to the window location and window width set at 40 and 350.For each CT sequence, regions of interest (ROIs) were manually delineated slice by slice by a radiologist with 15 years of experience using ITK-SNAP v3.6 (www.itksnap.org). The ROIs included both intra-tumoral and peri-tumoral regions. In all slices containing the primary lesion, the intra-tumoral delineation covered the entire tumor. The peri-tumoral region was annotated by the radiologist, including adjacent tissues and lymph nodes around the esophagus, excluding airways, aorta, vertebrae, and jugular vein. **Supplementary Figure** shows enhanced CT images and images after ROI delineation for two ESCC patients.

### Radiomics feature extraction

This study utilized the Pyradiomics software package in Python v3.6 to extract a total of 1967 features from the CT sequences of each patient. These features can be divided into 18 first-order features, 14 shape-based features, and 75 texture features. Texture features include 24 Grey Level Co-occurrence Matrix (GLCM) features, 16 Grey Level Run Length Matrix (GLRLM) features, 16 Grey Level Size Zone Matrix (GLSZM) features, 5 Neighboring Grey Tone Difference Matrix (NGTDM) features, and 14 Grey Level Dependence Matrix (GLDM) features. These features also include high-dimensional feature types, calculated from images transformed by 8 filters (including wavelet, Laplacian of Gaussian, square, square root, logarithm, exponential, gradient, and local binary pattern 2D filters). For a detailed description of the features and extraction protocol, please refer to the Pyradiomics documentation (https://pyradiomics.readthedocs.io).

### Feature selection

Feature selection was performed using R language (version: 3.6). Firstly, 30 patients were randomly selected, and a second ROI segmentation was performed on the CT images of the patients by another radiologist. The Intraclass Correlation Coefficient (ICC) was then used to assess the reliability of features extracted from ROIs delineated by different radiologists (19), with features having an ICC exceeding 0.80 considered more reliable and retained. Subsequently, the Mann-Whitney U test was used to screen the remaining features, a widely used non-parametric test method, to evaluate features extracted from intra-tumoral and peri-tumoral radiomic features separately or in combination for identifying GR, with features having P < 0.05 considered statistically significant and retained. Next, using the “glmnet” package in R language, features with non-zero coefficients were retained using the Least Absolute Shrinkage and Selection Operator (LASSO) algorithm with 10-fold cross-validation (20). Finally, the Akaike Information Criterion was used as the stopping criterion to perform logistic regression on the selected features.

### Model establishment

The most predictive features selected from enhanced CT were used to construct the radiomics model. For the selected features from enhanced CT, feature fusion was performed using LASSO coefficients to establish a multi-phase CT radiomics fusion model (Rad-Score). Clinically significant features were selected using the Mann-Whitney U test and chi-square test (*P* < 0.05), and a clinical prediction model was constructed using logistic regression. During model development, the training dataset was randomly split into five equal folds. The model was trained on four folds and validated on the remaining one, rotating until each fold had served as the internal validation set. Performance metrics, such as accuracy and area under the curve, were recorded and the averaged value of them was used as the final metrics to evaluate the model’s performance. Finally, the model’s performance was evaluated using the external validation set. The “rms” package in R language was used to combine Rad-Score with clinically significant features to construct a nomogram model.

### Model validation

IBM SPSS Statistics (version: 24) was used to conduct statistical analysis on all clinical features. To evaluate the correlation between intra-tumoral and peri-tumoral radiomic features and clinical features, the Mann-Whitney U test was performed for age, and the chi-square test was performed for gender, tumor location, pathological type, N stage, M stage, clinical stage, and number of chemotherapy cycles. The significance level was set at 0.05 for two-sided hypothesis testing.

To assess the predictive performance of the three models, including, Rad-Score, T-stage, and Nomogram, receiver operating characteristic (ROC) curve analysis was conducted on both the training and internal validation cohorts. ROC curves were plotted using the “pROC” package in R language, and the area under the ROC curve (AUC) was calculated. The Delong test was used to compare the differences between the ROC curves of each model (21). The maximum Youden index was used as the optimal threshold to calculate accuracy (ACC), specificity (SPE), and sensitivity (SEN). Calibration curve and decision curve analyses were performed using the “rmda” package in R to evaluate the clinical utility of the nomogram model (22, 23).

## Results

### Patients

This study included a total of 120 patients in the primary cohort, with 90 patients in the training cohorts and 30 patients in the internal validation cohorts, comprising 115 males and 5 females, All included patients were of Han Chinese ethnicity, the GR rate of the primary cohort was 43.3%. Additionally, 30 patients from another hospital were included as external validation cohort, with 27 males and 3 females, the external validation cohort GR rate of 46.7%. The mean (SD) age of all patients was 61.47 (7.04) years, the percentage of all patients with tumors located at proximal third was 16.0%, located at middle third was 52.67%, and located at distal third was 31.33%. Analyses based on Student’s t-test, the Mann–Whitney U test, the chi-square test, or Fisher’s exact test revealed that there was a significant difference in T stage between the GR and non-GR groups in the training cohort, internal validation cohort, and external validation cohort (training cohort *P*=0.035; internal validation cohort *P*=0.047; external validation cohort *P*=0.041). However, there were no significant differences between the GR and non-GR groups in terms of gender, age, tumor location, N stage, staging of distant metastasis, clinical stage, and number of chemotherapy cycles in the training cohort, internal validation cohort, and external validation cohort (*P*>0.05). The final model was built using the T stage as the predictor variable. The statistical analysis results of clinical features for all patients are presented in **Table 1**.

**Table 1:** Statistical analysis results of patient clinical features.

Characteristic	Training Cohort (n=90)		*P*	Internal Validation Cohort (n=30)		*P*	External Validation Cohort (n=30)		*P*
	non-GR (n=51)	GR (n=39)		non-GR (n=17)	GR (n=13)		non-GR (n=16)	GR (n=14)	
Sex			0.154			0.150			0.144
Male	49	38		17	11		15	12	
Female	2	1		0	2		1	2	
Age (Mean ± SD)	61.53 ± 7.40	61.80 ± 6.76	0.719	60.93 ± 7.14	59.47 ± 7.21	0.368	62.43 ± 6.14	61.87 ± 7.28	0.427
Tumor location			0.165			0.182			0.206
Proximal third	5	8		2	4		2	3	
Middle third	27	21		11	4		9	7	
Distal third	19	10		4	5		5	4	
Clinical T stage			0.035*			0.047*			0.041*
1	3	2		0	1		1	1	
2	8	5		3	1		2	4	
3	33	26		10	10		7	6	
4	7	6		4	1		6	3	
Clinical N stage			0.147			0.379			0.453
0	4	3		4	3		1	2	
1	39	30		12	8		11	6	
2	7	5		1	1		2	4	
3	1	1		0	1		2	2	
Staging of distant metastasis			0.522			0.695			0.697
0	35	26		12	11		12	13	
1	16	13		5	2		2	3	
Clinical stage group			0.107			<0.001*			0.052
I	3	5		0	1		1	0	
II	4	6		1	2		2	1	
III	37	23		12	6		10	9	
IV	7	5		4	4		3	4	
Number of treatment cycles			0.596			0.817			0.806
2	13	21		10	5		6	7	
2+	38	18		7	8		10	7	

* *P*<0.05.

### Development of the radiomics signature

**Figure 2:**
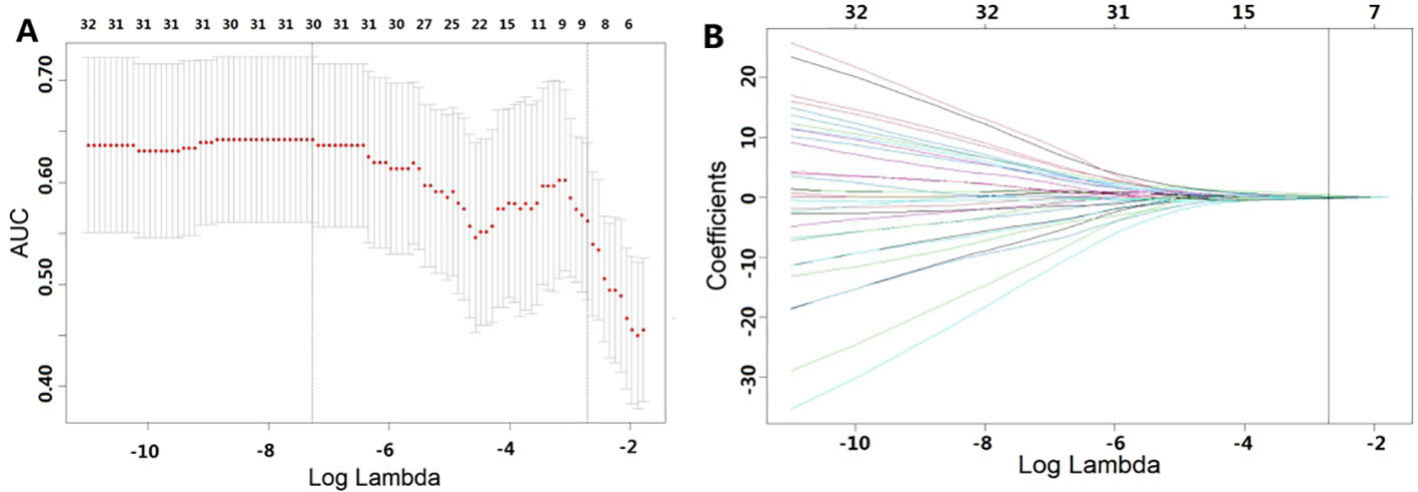
Features selected using LASSO. **(A)** represents the results of ten-fold cross-validation, where red dots denote the coefficients corresponding to each λ, and the dashed line indicates the specific λ values. **(B)** illustrates the LASSO coefficient plot, with each curve representing the trajectory of change in the coefficient of each independent variable, and the corresponding vertical axis represents the regression coefficient of the variable. **Table 2** presents the results of the radiomics feature selection, wherein 5 features were selected from the pool of 1967 features.

For the 1967 features extracted from enhanced CT, analysis was conducted using the Mann-Whitney U test, and logistic regression was applied with the AIC criterion as the stopping rule. The final selection identified 5 radiomic features as the most important predictive factors for constructing the radiomics model for predicting GR. The LASSO algorithm, used for feature selection, was visualized in **Figure 2**.

**Table 2:** The predictive performance of the ultimately selected radiomics features.

Feature	Cohort	Mean ± SD		*P*	AUC
		GR	non-GR		
lbp.2D_firstorder_ Median	Training	4.470 ± 0.507	4.200 ± 0.407	0.025	0.633
	Internal Validation	4.400 ± 0.507	4.200 ± 0.414	0.237	0.600
	External Validation	4.200 ± 0.414	4.200 ± 0.414	0.235	0.601
logarithm_glszm_ HighGrayLevel ZoneEmphasis	Training	295.088 ± 186.703	389.111 ± 156.683	0.031	0.656
	Internal Validation	365.573 ± 152.244	415.395 ± 150.685	0.530	0.569
	External Validation	374.278 ± 171.454	452.241 ± 157.627	0.587	0.574
wavelet.HHH_glszm_ SizeZoneNonUniformity	Training	17.955 ± 10.003	29.865 ± 21.880	0.015	0.674
	Internal Validation	21.718 ± 13.061	30.786 ± 16.798	0.081	0.678
	External Validation	19.255 ± 11.571	28.637 ± 18.918	0.074	0.671
wavelet.HLL_glcm_ MaximumProbability	Training	0.246 ± 0.036	0.229 ± 0.041	0.032	0.656
	Internal Validation	0.238 ± 0.033	0.209 ± 0.046	0.026	0.716
	External Validation	0.261 ± 0.045	0.187 ± 0.045	0.021	0.705
wavelet.LHH_glszm_ SmallAreaHighGray LevelEmphasis	Training	19.765 ± 7.750	26.685 ± 13.441	0.022	0.664
	Internal Validation	20.236 ± 8.538	24.950 ± 10.926	0.218	0.631
	External Validation	20.026 ± 8.736	25.869 ± 12.147	0.201	0.549

The fusion of the final selected features is used to construct the radiomics label Rad-Score for enhanced CT imaging. The formula is as follows:


$$\begin{array}{l} \text{Rad}-\text{Score}=-12.755-0.033\times \text{wavelet}.\text{HHH}\_\text{glszm}\_\text{Size}\text{ZoneNonUniformity}-2.654\\ \times \text{original}\_\text{firstorder}\_\text{Median}+18.982\times \text{wavelet}.\text{HLL}\_\text{glcm}\_\text{MaximumProbability}-0.003\times \text{logarithm}\_\text{glszm}\_\text{HighGrayLevelZoneEmphasis}-0.049\times \text{wavelet}.\text{LHH}\_\text{glszm}\_\text{SmallAreaHighGratLevelEmphasis}. \end{array}$$

**Figure 3:**
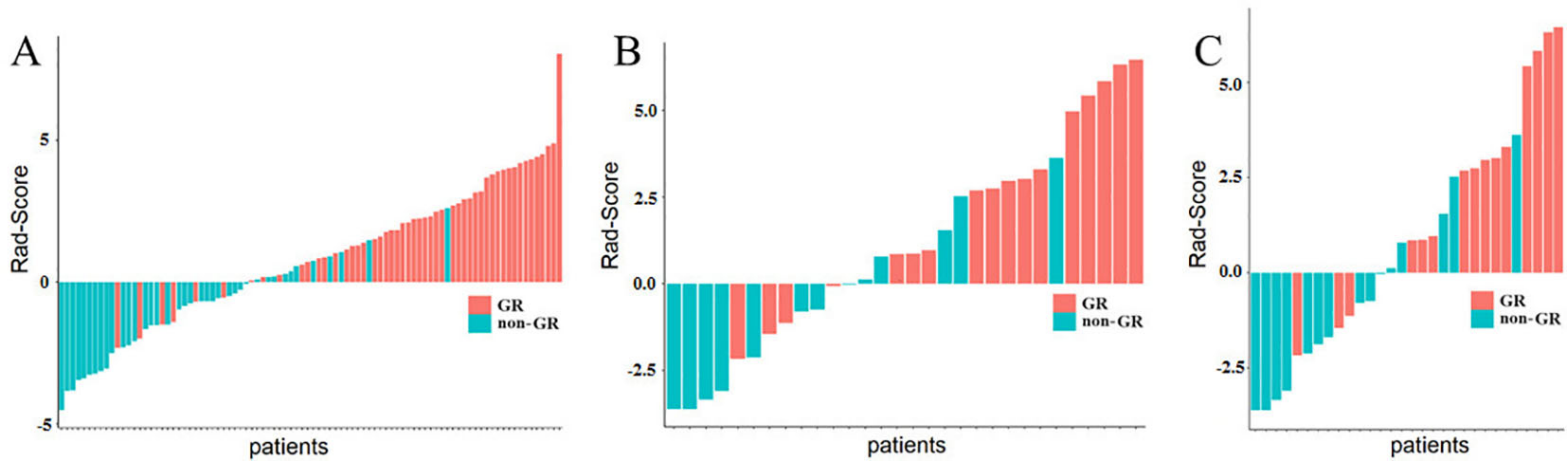
Rad-Score of ESCC Patients. **(A)** represents the Rad-Score of each patient in the training cohort, **(B)** represents the Rad-Score of each patient in the internal validation cohort. while **(C)** represents the Rad-Score of each patient in the external validation cohort. The red bars indicate GR patients, while the green bars indicate non-GR patients.

**Figure 3** displays the results of Rad-Score in distinguishing GR patients from non-GR patients. The study indicates that the Rad-Score can accurately differentiate between the majority of patients with GR and non-GR.

### Construction and evaluation of the nomogram

**Figure 4:**
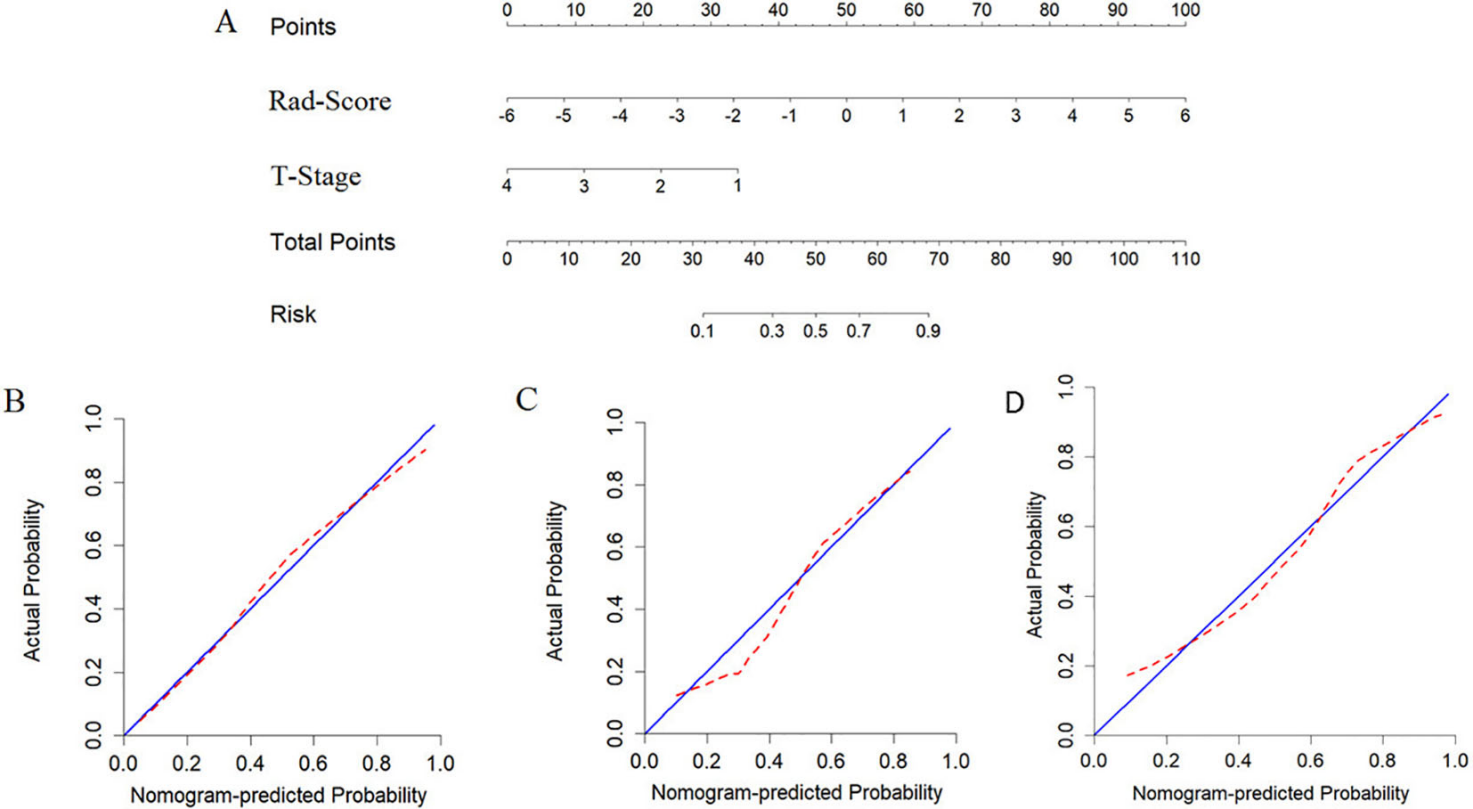
Developing nomograms to predict the GR status of ESCC patients. **(A)** represents the developed nomogram. **(B)** represents the calibration curve of the nomogram on the training cohort, **(C)** represents the calibration curve of the nomogram on the internal validation cohort. and **(D)** represents the calibration curve of the nomogram on the external validation cohort.

**Figure 5:**
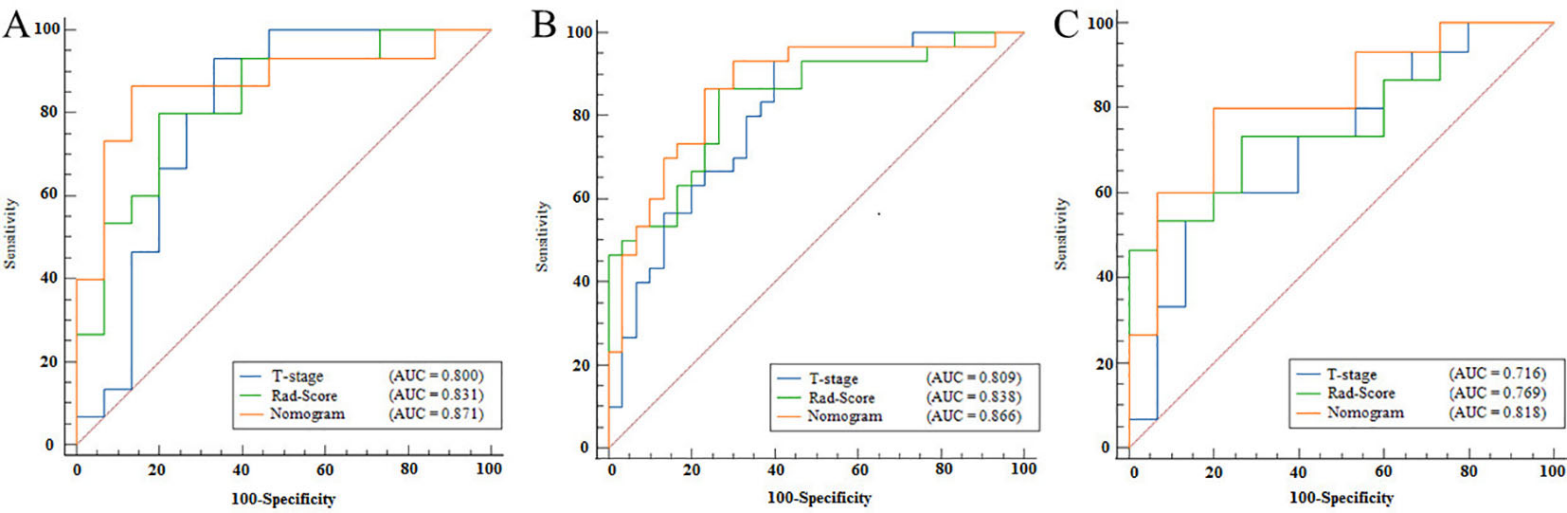
ROC curves for Rad-Score, T-stage, and nomogram. **(A)** represents the ROC curve of the training cohort, **(B)** represents the ROC curve of the internal validation cohort, and **(C)** represents the ROC curve of the external validation cohort. The horizontal axis in the figure represents the value of 100-specificity, and the vertical axis represents the value of sensitivity. The blue line represents the Rad-Score model, the green line represents the T-stage, and the red line represents the nomogram.

**Figure 6:**
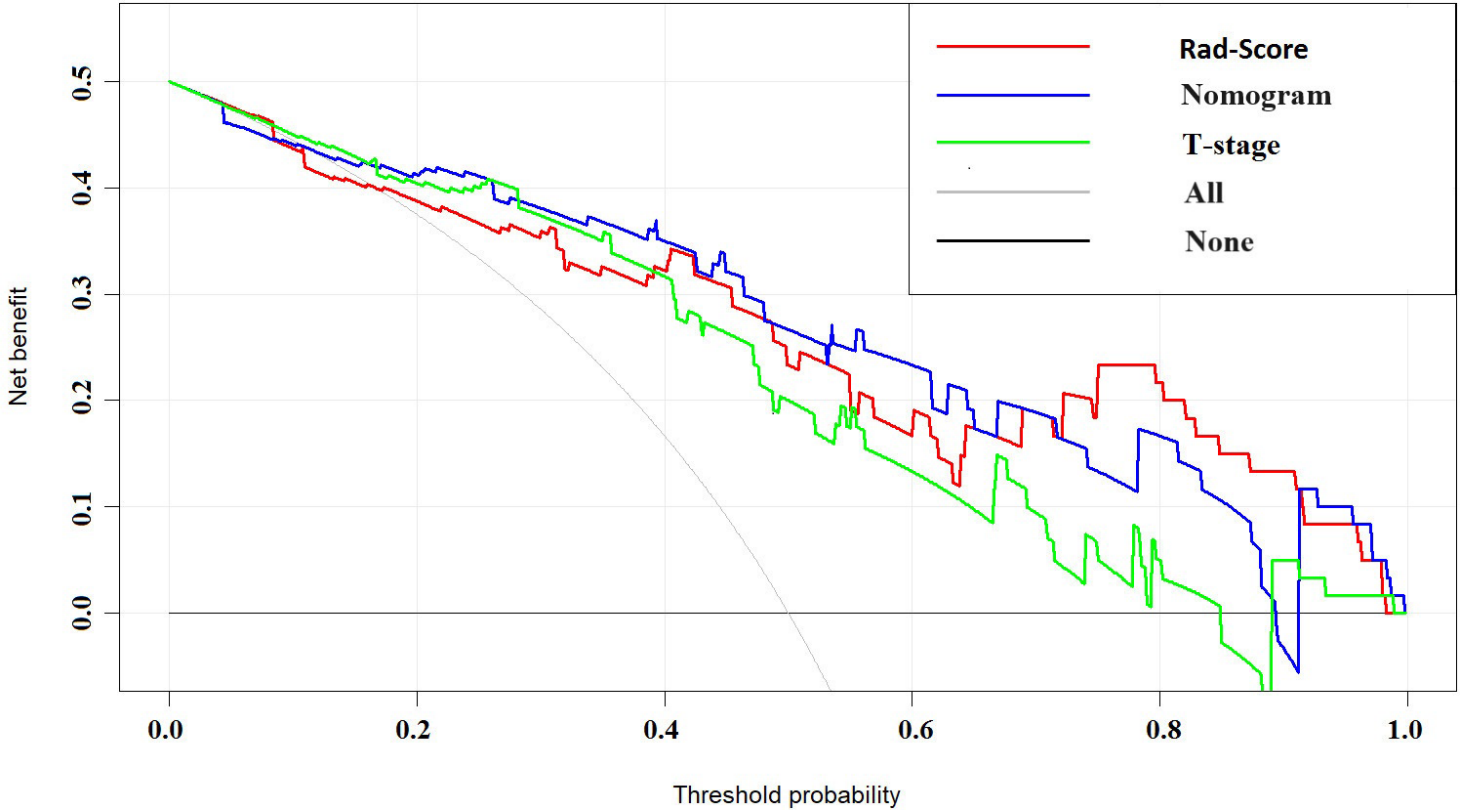
DCA curves of Rad-Score, T-stage, and nomogram. The horizontal axis in the figure represents threshold probability, while the vertical axis represents patients’ net benefit. The blue line represents the Rad-Score model, the green line represents the T-stage, and the red line represents the nomogram.

The nomogram model, integrating Rad-Score and T-stage, was constructed as depicted in **Figure 4**. Calibration curve results indicated that the predictive values of the nomogram model aligned well with the actual outcomes. **Table 3** evaluated and compared the predictive performance of Rad-Score, T-stage, and the nomogram. In both the training and internal validation cohorts, Rad-Score exhibited higher AUC and SEN compared to the T-stage. The AUC of the nomogram outperformed both Rad-Score and the T-stage (AUC in the training cohort: nomogram vs. Rad-Score vs. T-stage = 0.867 vs. 0.838 vs. 0.809; AUC in the internal validation cohort: nomogram vs. Rad-Score vs. T-stage = 0.871 vs. 0.831 vs. 0.800; AUC in the external validation cohort: nomogram vs. Rad-Score vs. T-stage = 0.818 vs. 0.769 vs. 0.716). **Figure 5** illustrates the ROC curves of each model. The decision curve analysis (DCA) (**Figure 6**) demonstrated a favorable net benefit of the nomogram in predicting GR. ESCC patients could benefit more when the threshold probability is between 0.08 and 0.85.

**Table 3:** Comparison of Rad-Score, T-stage, and nomogram.

Radiomics model	Training				*P*	Internal Validation				*P*	External Validation				*P*
	AUC	ACC	SPE	SEN		AUC	ACC	SPE	SEN		AUC	ACC	SPE	SEN	
T-stage	0.809	0.683	0.967	0.567		0.800	0.700	0.933	0.667		0.716	0.697	0.857	0.605	
Rad-Score	0.838	0.700	0.867	0.733		0.831	0.700	0.800	0.800		0.769	0.775	0.813	0.773	
Nomogram	0.867	0.733	0.900	0.733		0.871	0.667	0.867	0.867		0.818	0.795	0.830	0.870	
T-stage vs. Rad-Score					<0.001*					0.037*					0.028*
T-stage vs. nomogram					0.002*					0.022*					0.019*
Rad-Score vs. nomogram					0.005*					0.596					0.116

* *P*<0.05.

## Discussion

Predicting the efficacy of neoadjuvant therapy in ESCC patients is crucial for the development of personalized treatments. Although previous studies have attempted to predict the efficacy of treatments for certain diseases using biomarkers, there are currently no definitive biomarkers for immunotherapy in ESCC (24–26). Research has shown that utilizing tumor imaging data can provide more direct insights (16). Considering the accessibility and health economics of examinations, as a recommended method for preoperative tumor staging in esophageal cancer treatment guidelines (27), enhanced CT scans are regarded as a valuable tool. They can identify tumor changes, facilitate preoperative assessments, and predict treatment efficacy. This approach offers a macroscopic and direct method to assess tumor characteristics in patients with ESCC. Previous radiomics studies have only analyzed the intra-tumoral characteristics of ESCC patients, However, features should not be limited to the sole internal region of the tumor. Recent research (28–30) suggests that surrounding areas can provide complementary information on tumor heterogeneity. Therefore, we propose a radiomics model based on enhanced CT images, which combines intra-tumoral and peri-tumoral radiomics features to predict the efficacy assessment of ESCC patients after receiving NICT.

In this study, we comprehensively analyzed 1967 radiomics features extracted from intra-tumoral and peri-tumoral regions obtained from enhanced CT images. Subsequently, we screened out image features that can predict the efficacy of neoadjuvant therapy in esophageal cancer patients. Ultimately, we identified five most significant features from both intra-tumoral and peri-tumoral regions. Three features belonged to gray-level size zone matrix (GLSZM) features. GLSZM quantifies regions of gray levels in images, defined as the number of connected voxels sharing the same gray level intensity. One feature belonged to first-order features, describing voxel intensity distribution within the image region defined by a mask. Another feature belonged to gray-level co-occurrence matrix (GLCM), a method for describing texture features by studying the correlation of different gray values at specific angles and distances within an image. The classification results of these features suggest a potential correlation between patient GR state and tumor heterogeneity, as features based on first-order statistics, GLCM, GLSZM, neighborhood gray-tone difference matrix (NGTDM), gray-level run length matrix (GLRLM), and gray-level dependence matrix (GLDM) are generally considered to reflect tumor heterogeneity at both global and local scales (31).

Wu et al. (32) previously, extracted 10 intra-tumoral features from CT images of 154 patients for analysis. Results showed that some features could differentiate early (Stages I-II) and advanced (Stages III-IV) ESCC, with respective training cohort areas under the receiver operating characteristic curve (AUC) of 0.795 and 0.694, and internal validation cohort AUCs of 0.762 and 0.624. In contrast, our results showed that combining intra-tumoral and peri-tumoral tissue in the predictive model yielded AUC values of 0.809 and 0.800 on the training and internal validation cohorts, respectively, this suggests that the peri-tumoral region may provide complementary useful information, thereby potentially enhancing the predictive ability of the model. This finding is partially consistent with recent research results (33–36). Radiomics features, particularly those derived from texture analysis (e.g., Gray Level Size Zone Matrix [GLSZM], Gray Level Co-occurrence Matrix [GLCM], and first-order statistical features), have been extensively studied as imaging biomarkers reflecting tumor heterogeneity, which is closely associated with therapeutic response and prognosis. The features selected in this study underwent a rigorous statistical screening process (including LASSO regression) to ensure their predictive validity. While radiomics features are inherently data-driven and may not always directly correlate with specific histopathological markers, prior studies suggest that texture-based metrics can reflect tumor microenvironment characteristics such as necrosis, fibrosis, and angiogenesis.

Dong et al. (37) and Liu et al. (38)previously demonstrated the importance of clinical T-stage as a predictive indicator. Through statistical analysis of clinical features, we also found that T-stage is an important predictor of GR status, negatively correlated with GR status in ESCC patients. The AUC for T-stage was 0.809, 0.800, and 0.716 for the training cohort, internal validation cohort, and external validation cohort respectively. The Rad-Score for predicting response to NICT achieved an AUC of 0.838 for the training cohort, 0.831 for the internal validation cohort and 0.831 for the external validation cohort. By combining T-stage with the established Rad-Score model, we established a clinical imaging radiomics nomogram, which exhibited the best predictive performance. the AUC values for the training cohort, internal validation cohort, and external validation cohort were 0.867, 0.871, and 0.818 respectively. Outperforming both standalone radiomics and clinical T-stage models, indicating improved ability to detect GR in patients. DCA indicated that our nomogram model could provide more benefits for ESCC patients in predicting GR. The visual approach of the nomogram compared to machine learning algorithms may assist doctors in diagnostic decision-making. Predictive models can non-invasively and accurately predict the efficacy of NICT, providing valuable choices and suggestions for personalized treatment of ESCC patients, thus alleviating the burden and suffering of patients during treatment and holding significance for prognosis and treatment prediction.

In our study, we adopted LASSO regression due to its ability to effectively handle high-dimensional data and perform feature selection by setting the coefficients of certain features to zero, thereby enhancing the interpretability of the model. In contrast, other models like Random Forest and XGBoost, which rely on ensembles of decision trees, lack intuitive transparency in explaining how predictions are derived. Our choice of LASSO regression was driven by the need for interpretable results and clinical applicability in real-world settings. Studies by other scholars (39, 40)have also use LASSO regression to selected Radiomics features, demonstrating the efficacy of machine learning in radiomics-based survival prediction and its significant clinical value.

This study has limitations. Firstly, it is a retrospective study with a relatively small sample size. In the future, using more clinical data to validate our model could enhance its robustness. Secondly, the heterogeneity of patients leads to different responses to treatment. Although we made effort to ensure consistency in patient during initial screening, including ethnicity, treatment protocols, and pathological type. heterogeneity factors such as variations living environments, histories of smoking or alcohol consumption were not fully accounted for. These factors may act as additional confounding variables in predicting therapeutic efficacy, necessitating large datasets and multicenter studies to address these complexities. Thirdly, the manual delineation of ROI introduces variability despite the involvement of radiologists, leading to poor repeatability of imaging data and time-consuming operations. Incorporating deep learning-based lesion automatic or semi-automatic segmentation holds promise for improving this aspect in our future work. Thirdly, there is a lack of follow-up data regarding patients’ survival time, which hinders the analysis of subsequent features and parameters.

## Conclusion

The results indicate that the model based on radiomic features from both intra-tumoral and peri-tumoral regions on enhanced CT imaging, combined with clinical T-stage features, exhibits promising predictive potential for assessing the efficacy of NICT in ESCC patients.

## Data Availability

The raw data supporting the conclusions of this article will be made available by the authors, without undue reservation.

## Supplementary material

Supplementary Figure Examples of enhanced CT images and ROI delineation regions in ESCC patients in this study. **(A)** represents the original image of a patient with GR, **(B)** represents the intra-tumoral image of a patient with GR, and **(C)** represents the peri-tumoral image of a patient with GR. **(D)** represents the original image of a patient with non-GR, **(E)** represents the intra-tumoral image of a patient with non-GR, and **(F)** represents the peri-tumoral image of a patient with non-GR. The red areas in the images represent the ROI. No significant differences were observed upon visual inspection.
