# Supplementary figures and images for: CT-based radiomics for prediction of response to neoadjuvant immunochemotherapy in patients with esophageal carcinoma
Source: Front Oncol. 2025 May 12;15:1511691. doi: 10.3389/fonc.2025.1511691 (PMC12163236; doi:10.3389/fonc.2025.1511691)

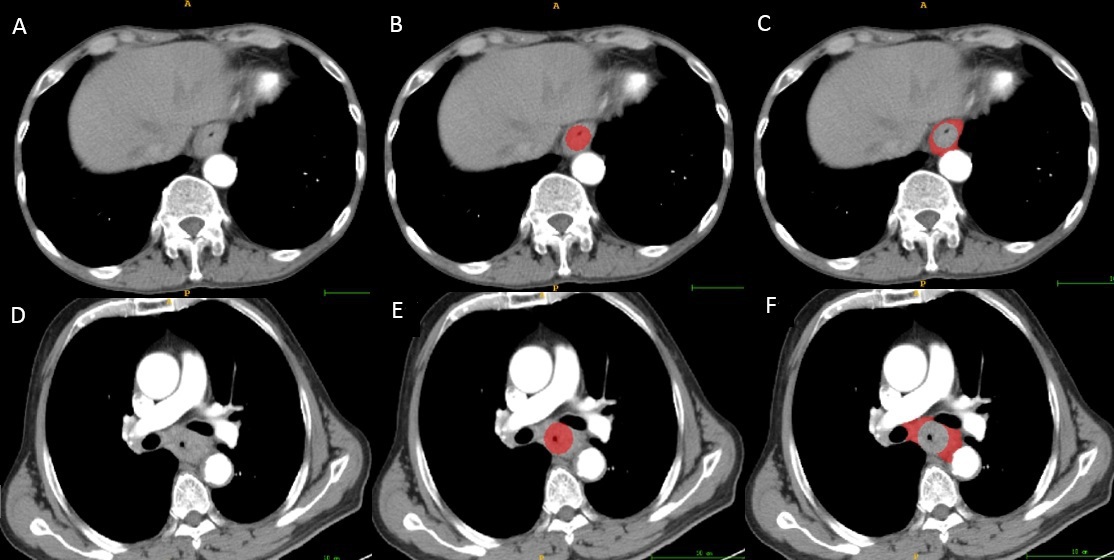

Supplement: Supplementary Figure — Examples of enhanced CT images and ROI delineation regions in ESCC patients in this study. (A) represents the original image of a patient with GR, (B) represents the intra-tumoral image of a patient with GR, and (C) represents the peri-tumoral image of a patient with GR. (D) represents the original image of a patient with non-GR, (E) represents the intra-tumoral image of a patient with non-GR, and (F) represents the peri-tumoral image of a patient with non-GR. The red areas in the images represent the ROI. No significant differences were observed upon visual inspection. [file Image1.jpeg]
